# Molecular insight into COF monolayers for urea sorption in artificial kidneys

**DOI:** 10.1038/s41598-021-91617-1

**Published:** 2021-06-08

**Authors:** Ahmad Miri Jahromi, Mohammad Khedri, Mehdi Ghasemi, Sina Omrani, Reza Maleki, Nima Rezaei

**Affiliations:** 1 Computational Biology and Chemistry Group (CBCG), Universal Scientific Education and Research Network (USERN), Tehran, Iran; 2 Department of Petroleum Engineering, Amirkabir University of Technology, Tehran, Iran; 3 Department of Petroleum Engineering, University of Tehran, Tehran, Iran; 4 Research Center for Immunodeficiencies, Pediatrics Center of Excellence, Children’s Medical Center, Tehran University of Medical Sciences, Tehran, Iran; 5 Network of Immunity in Infection, Malignancy and Autoimmunity (NIIMA), Universal Scientific Education and Research Network (USERN), Tehran, Iran; 6 Department of Immunology, School of Medicine, Tehran University of Medical Sciences, Tehran, Iran

**Keywords:** Biochemistry, Diseases, Materials science

## Abstract

Urea removal from an aqueous solution is considered a challenge in the biological process. The state of complete kidney destruction is known as an end-stage renal disease (ESRD). Kidney transplant and hemodialysis are the most common methods for confronting ESRD. More recently, wearable artificial kidney (WAK) devices have shown a significant improvement in urea removal performance. However, low efficiency in physical adsorbents is a barrier in developing them. For the first time, the urea adsorption capacity of five types of last-generation covalent organic framework (COF) nanosheets (NSs) was investigated in this study by applying molecular dynamics (MD) simulation tools. To this end, different analyses have been performed to evaluate the performance of each nanoparticle. The MD all-atom (AA) results demonstrated that all introduced COF NSs had urea removal capacity. Among the five NSs, TPA-COF was shown to have the best outcomes. Moreover, coarse-grained (CG) and density functional theory (DFT) simulations were conducted, and the results show that the TPA-COF nanoparticle modified with –OH functional group has even better properties for urea adsorption. The present molecular study sheds new light on COF NSs as an adsorbent for urea removal.

## Introduction

Urea has a vital role in industrial and biological processes, and it is the main by-product of protein metabolism. It was reported that 84% of urine and 68% of the sweat is built from urea^1,2^. The human body produces 20–30 g of urea per day^3^. It is the most significant product of protein metabolism in the human body. Urea is transported to the kidneys and excreted into the urine, and a high concentration of urea is an indicator of liver and kidney function^4^. Acute kidney injury and chronic kidney disease (CKD) will accumulate urea or uremia. Hyperuremia is a condition of having high levels of urea in the blood due to kidney failure and can result in organ failure and death if left untreated. Uremia causes a wide variety of clinical features since uremia affects the function of many organs and systems. CKD is a progressive and irrecoverable disease that, at its final stage, will function of both kidneys will be lost. This stage is called end-stage renal disease (ESRD). To date, most of the treatments for managing this condition are focused on slowing down this process^5^. In the blood of a uremic patient, the urea compound has the highest concentration, and it is a predominant component among the toxins in the blood. As a result, effective urea removal is crucial for successful dialysate^6,7^.

Kidney transplant is the primary method for curing ESRD and around 20,000 kidney transplant was performed in 2016 in the US. Unfortunately, it seems that the number of kidney transplantation candidates is increasing much faster than the number of kidney donors^8^. Due to the lack of kidney donors and transplant cost, hemodialysis and hemoperfusion are still the regular treatment of uremia. A functional kidney works 168 h per week, but a patient with CKD will receive 9–12 h of dialysis per week because of difficulties in the utilizing dialysate machines at hospitals or clinics. Patients suffering from CKD need to be connected to the device in which each one takes a couple of hours to purify the blood, and it possesses high energy consumption^9^. Furthermore, the traveling time to the health center and restrictions on patient diets make the procedure much more exhausting since the waste removal capacity of these devices are still far from a healthy kidney. Therefore, researches have been developed to find a more efficient alternative way for blood waste disposal. In recent past years, wearable artificial kidney (WAK) device have undergone lots of theoretical and experimental investigations and have shown a potential for changing the cure of ESRD toward a much convenient procedure. A wearable device provides patients with the ability to move around and perform every activities. Furthermore, it works during the day and decreases the pace of fluid and urea removal, and reduces interdialytic symptoms. A WAK converts urea to ammonia, which leads to the generation of carbon dioxide that can disrupt the flow in the circuit. This phenomenon can be prevented by a gas-permeable section between the sorbents and the dialyzer^10^. Among the practical application of WAKs, there are some minor problems that can be reformed by redesigning. The most prominent issue in WAKs is the low urea removal efficiency of applied methodology in WAKs, and still, the quest for finding reliable methods continues.

According to the literature, there are many urea removal methods divided into two main categories: (1) non-electrochemical processes such as hydrolysis, decomposition in the biological bed, enzymatic hydrolysis, decomposition by strong oxidants, adsorption, catalytic decomposition, and (2) electrochemical methods. The fact is that some of these urea removal techniques are not either efficient or cost-effective^11^. Thus, some of these methods are still new and require more research and optimization because of equipment complexity or other industrial inapplicability^12^. One of the critical factors in urea removal methods, especially in hemodialysis, is the amount of required time to accomplish the process. Indeed, optimizing a technique in which more urea molecules are adsorbed in less time can be a real help for increasing the quality of life for the patients. The inadequacy of current conventional methods leads to high mortality and low quality of life^13^. Among non-electrochemical methods, hydrolysis of urea showed the best effectiveness^12^. However, the required high pressure and temperature conditions during the process would be a drawback in industrial usage^14^. Also, urea removal by biological methods has much complexity, and reaching the best operation condition requires further researches^15,16^. The enzymatic decomposition of urea is considered an effective way that can be much faster than the unanalyzed urea hydrolysis, however, finding a stable enzyme is a challenge. Decomposition by strong oxidants requires finding a non-toxic and cheap oxidant that doesn’t produce toxic materials after urea decomposition. Among all available urea removal methods, it can be said that the adsorption method is more promising, nevertheless, finding the most effective adsorbent and optimization of process condition is still an issue^12^.

The adsorption process can be considered as a cost-effective and straightforward technique among other urea removal methods. It would be an attractive method for urea removal for both water purification and human body purposes. There will be no hazardous side-product and can increase purification capacity^7,17^. The process of urea adsorption consists of flowing the solution through a sorbent bed, but a usual problem with this process is low efficiency. Numerous materials have been introduced as urea sorbents, such as activated carbons, zeolites, montmorillonite, chitosan, MXene, oxystarch, etc.^18–21^. The fact is that low adsorption capacity, toxic effect, or even bio incompatibility reduce these adsorbents' efficiency. Although, some of the barriers reducing the performance of adsorbents can be eliminated, such as human incompatibility limitation that can be solved by using a semipermeable membrane that separates urea sorbent and blood^7^. These drawbacks restrict the use of sorbent materials for urea adsorption, and the search for a new alternative still is a requirement^11^. A suitable urea sorbent would be able to form covalent and non-covalent bonds with urea in a temperature range of the human body proceeding into its removal from the patient’s body^13^. Finding an efficient urea sorbent in the aquatic environment is still a challenge because the alteration of the surface hydrophobicity/hydrophilicity or using sorbents with narrow pores cannot improve sorbent selectivity for adsorption of urea^20^. One of the problems is the similarity between water and urea that makes them compete for sorbents' adsorption sites. There have been some experimental measurements to characterize different sorbents' properties^22,23^. The fact is that, none of the currently available urea removal sorbents make a truly wearable dialysis devices efficient. Further research should focus on introducing more effective sorbents so as to make WAK device feasible.

In recent years, covalent organic frameworks (COFs) have got attention due to having particular characteristics. COFs are built by the light elements (such as H, B, C, N, O) connected through strong covalent bonds that first time were reported by Cote et al.^24,25^. COFs have remarkable properties of high surface area, robust stability, lightweight, chemical stability, low density, etc.^26–28^. COFs are designable and controllable materials, and the main reason that separates these structures from other materials is their capability of interactions and trapping. Regarding these properties, COFs have practical uses in gas storage, energy storage, catalysis, drug delivery, gas adsorption, etc.^22,29–31^.

In the present study, with the use of molecular simulations including all-atoms (AA), coarse grain (CG), and density functional theory (DFT) as powerful techniques that provide both qualitative and quantitative insights on the identification of physical and chemical interaction mechanisms of biological systems, for the first time, the urea removal efficiency of five types of last-generation covalent organic framework (COF) nanosheets (NSs) including TPA-COF, DAAQ-TFP, DAPH-TFP, Tp-PaSO_3_Li-COF, and PhOS-COF-1 was investigated by AA simulations. In the next step, the most effective COFs of AA simulations and TPA-COF modified with –OH functional group were selected for further investigation through CG and DFT simulations.

## Results

### All atom (AA) simulations

The position of particles at the beginning and end of the simulation of urea molecules adsorption by the aforementioned sorbents are shown in Supplementary Fig. S1 (Fig. 1 of supplementary) To compare urea molecules adsorption capability of COF nanoparticles in WAK devices, nine different analyses have been assessed. The evolution of all analyses provides insight on molecular system stability, process spontaneity, interactions between urea molecules and adsorbent, and distribution of urea molecules around adsorbent. Analyses that have been applied in this simulation are as follows.

#### Evaluating the type of interactions: interaction energy

This analysis measures absolute values of Van der Walls (VdW) energy and electrostatic forces in the interaction between urea molecules and COF adsorbent.

**Figure 1:**
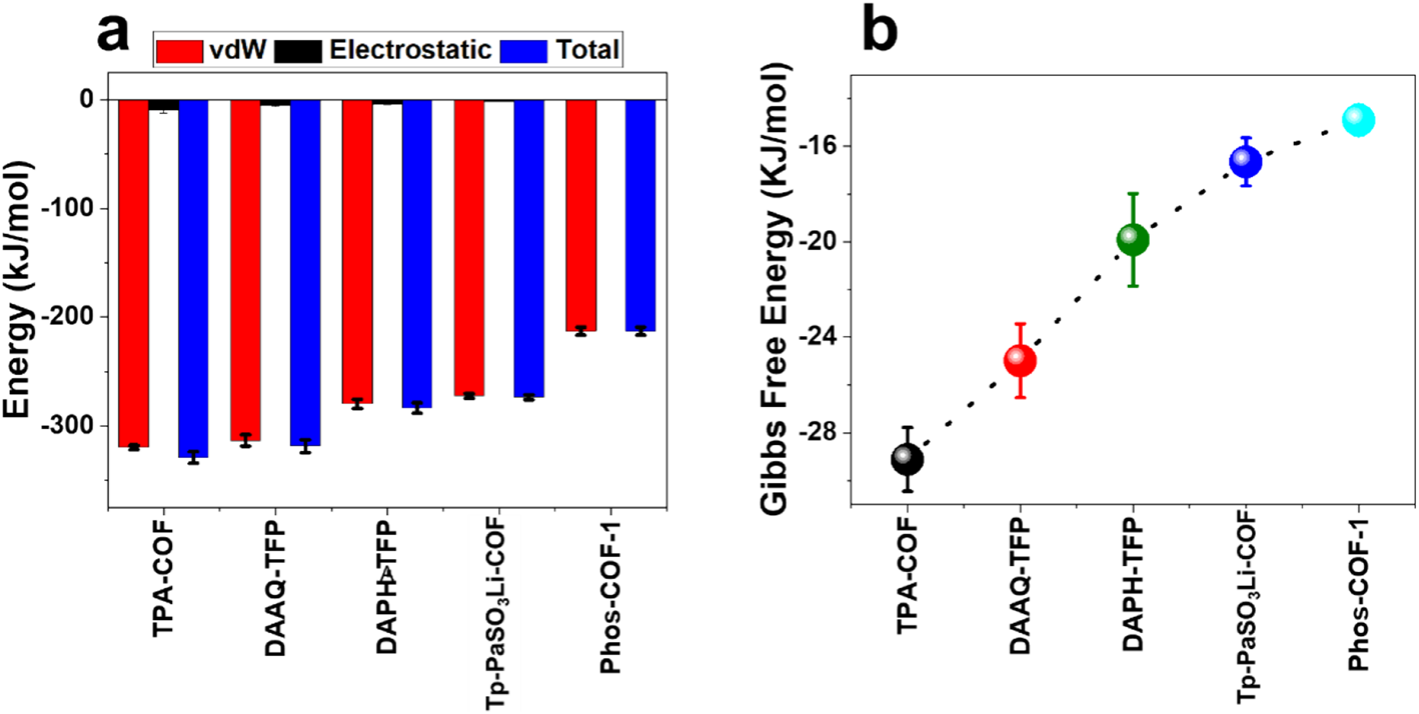
(**a**) Interaction energy between urea molecules (**b**) Gibbs free energy.

Regarding the interaction energy analysis, the more negative energy values, the more attractive forces between urea molecules and nanoparticles will result. Distance reduction between urea molecules and adsorbents resulting from high attractive forces demonstrates more stability^32^. Figure 1a presents the average interaction energies of simulated systems generated by the VdW and electrostatic bonds. As seen, the generated energy as a result of electrostatic interaction between urea molecules and adsorbents is approximately equal to zero. This can be ascribed to the absence of the charge for involved particles of the simulation system. In other words, the electrostatic interaction had no role in the adsorption of urea molecules on the nanoparticles. While dominant driving force during the adsorption of urea molecules on the adsorbents is VdW interaction, which is equal to the total interaction energy. It is shown that negative energy values reveal the attractive force of the urea molecules towards adsorbents in all simulations. Among all absorbents, as simulation proceeds, the total interaction energy of the TPA-COF experienced monotonically changes, which is an indication of more stability during the adsorption process. As was mentioned, the electrostatic bonds of systems are near zero. However, the order of significance of VdW interactions between urea molecules and adsorbents is as follows: TPA-COF > DAAQ-TFP > DAPH-TFP > Tp-PaSO3Li-COF > PHOS-COF. The fact is that the average value of total energy is considered a primitive indication of the level of interactions between particles.

#### Insights into the possibility and stability of process: Gibbs free energy

Gibbs free energy is the best method for determining the stability of any given complex system. The umbrella sampling technique has been conducted on all nanoparticles to calculate the Gibbs free energy by applying similar previous research procedures^33^.

The fact is that negative Gibbs free energy changes for a process are an indicator of a thermodynamically possible process, and also analyzing Gibbs free energy can determine the stability of a process. As shown in Fig. 1b, Gibbs free energy was negative for all nanoparticles indicating that all of these nanoparticles can be utilized in artificial kidneys to remove urea molecules. The lowest Gibbs free energy was observed in the TPA-COF nanoparticle, indicating the highest amount of urea molecules adsorption on the nanoparticle. Furthermore, the lowest Gibbs free energy specifies the most stable urea molecules adsorption. Consequently, the greatest performance of the adsorbent in regard to adsorption and stability is related to the TPA-COF nanoparticle. These results are consistent with the interaction energy analysis.

#### Analyzing the degree of attraction: the number of hydrogen bonds

**Figure 2:**
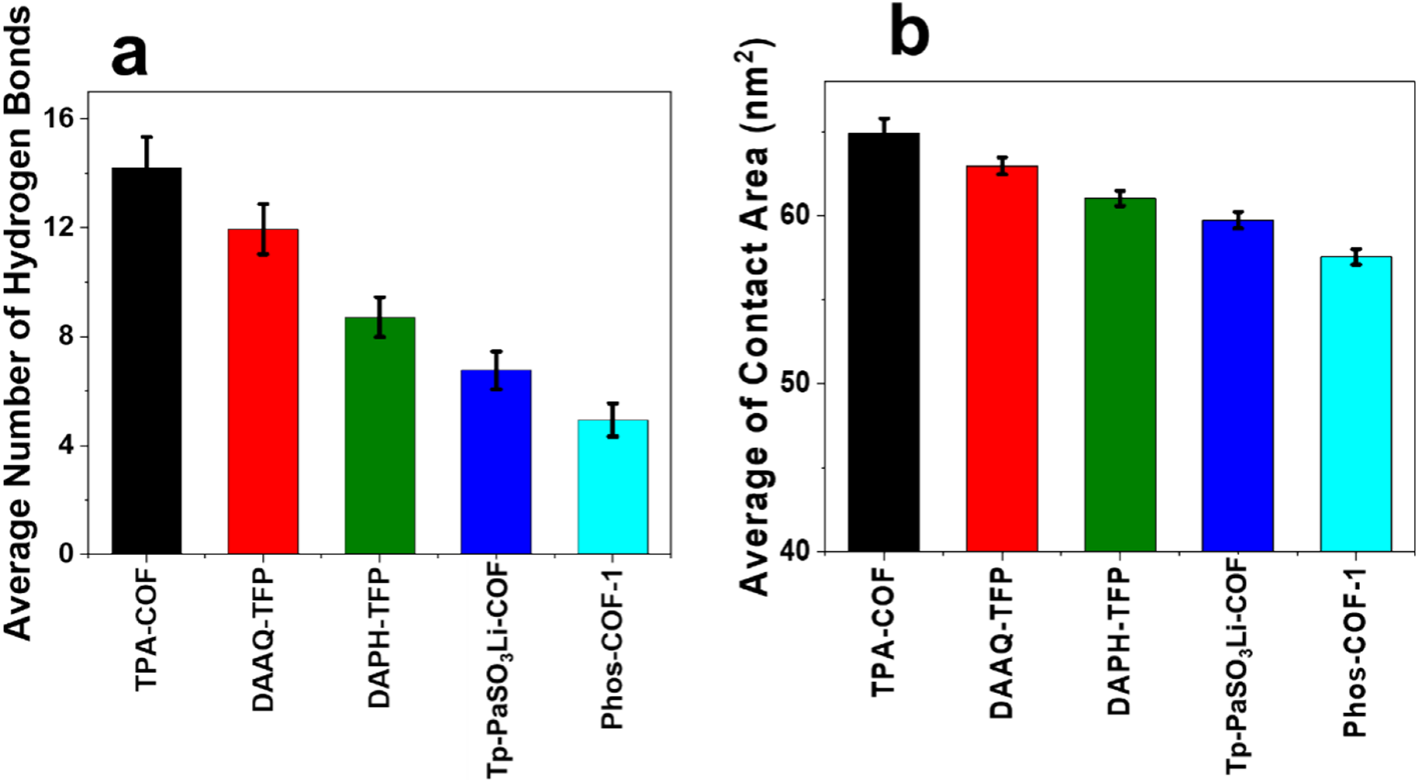
(**a**) Average number of hydrogen bonds between urea molecules and COFs Nanoparticles (**b**) Average of contact area.

Interaction between hydrogen atoms and high electronegative atoms such as oxygen, nitrogen, and fluorine can form hydrogen bonds. In biological systems, hydrogen bonds can be made due to interactions between hydrogen atoms and various electronegative biomolecules. In this analysis, an increase in the number of hydrogen bonds between urea molecules and nanoparticles can be considered an index of more attraction and higher system stability. In more detail, urea removal with sorbents is either by forming covalent bonds (chemisorption) or by non-covalent bonds (physisorption). Physisorption is much faster and reversible in comparison with the chemisorption process^7^. The fact is that urea is a polar molecule and non-covalent bonds mainly occur via hydrogen bonding and dipole interaction. Since the urea adsorption on the surface of the structures does not form covalent bonds between the urea and the nanostructures, and also the urea bonds are not broken, the process of urea adsorption in this work has been physisorption. On the other hand, an amine functional group of urea molecules can form hydrogen bonds with adsorbents in the case of an appropriate condition. Although one of the most important factors in creating a hydrogen bond is the presence of electronegative atoms such as oxygen, nitrogen, and fluoride in the structure of materials. But in addition to the existence of these atoms, it is necessary that the spatial coordinates of the molecular structures be such that the atoms are placed next to each other at a suitable angle. Also, atoms must be at a suitable distance from each other to form a hydrogen bond. The angle required for the formation of hydrogen bonds was less than 30°, and also the distance between the atoms should be less than 3.5 Angstrom. Figure 2a shows the average number of hydrogen bonds. In general comparison, it is clear that all of the simulated adsorbents could form hydrogen bonds. In this regard, the interactions between urea and TPA-COF have been more than the interactions between DAAQ-TPA, DAPH-TPA, and urea. So, these interactions caused the suitable spatial position to form more hydrogen bonds between urea and TPA-COF. On the other hand, DAAQ-TPA, DAPH-TPA structures had a larger surface than TPA-COF. In this work, the hydrogen bonds created between them are normalized to compare the intensity of urea adsorption by different structures. In fact, due to the size of the TPA-COF surface, the hydrogen bonds of TPA-COF and urea have been more. Although the hydrogen bonds are less than the number of nitrogen atoms in the TPA-COF structure, the hydrogen bonds created between urea and TPA-COF are not limited to the bond between the nitrogen of TPA-COF and hydrogen of urea because hydrogen bond is also created between the hydrogen of TPA-COF and the nitrogen and oxygen of urea molecules. As a result, urea molecules can form more than ten hydrogen bonds with TPA-COF. To compare the ability of different nanoparticles to adsorb urea molecules, the greatest number of H-bonds was formed between TPA-COF adsorbent and urea molecules, which indicated the efficient performance of the adsorbent in WAK devices.

#### Investigating the possibility of adsorption: SASA (solvent accessible surface area)

The self-assembly of particles can be demonstrated by SASA analysis. It is worth noting that the contact area analysis should be considered in parallel with other analyses since the contact area value can only reveal the self-assembly of particles, and this accumulation can be the result of adsorption or repulsion of nanoparticles. However, in this study, the higher amount of contact area specifies more accumulation of urea molecules around nanoparticles indicating more significant urea molecules adsorption^34,35^. Figure 2b shows the average contact area of urea molecules during the simulations. Increasing the contact area between urea molecules provides favorable conditions for the interaction of the particles. The results demonstrate that the system, which includes TPA-COF adsorbents, has a maximum average contact area. Thus, due to more conducive conditions for the particles' interactions, the accumulation of urea molecules around the nanoparticle increases.

#### Inspecting the size of nanoparticles: gyration radius

The radius of gyration (${R}_{g}$) is defined as the root mean square distance of each molecule from its center of mass in which its values provide precise and expressive information on the size of particles in comparison of end to end distance^36–38^.

To obtain information about the structure of a system, the degree of the molecule aggregation or dispersion in a molecular system can be monitored through the calculation of ${R}_{g}$. Regarding the interpretation of this analysis, the more stable and compacted system is obtained when the decrease of ${R}_{g}$ is higher. It is worth noting that the ${R}_{g}$ can reflects not only stability of system but also the intensity of energy between particles. In other words, considering the adsorption energy between the particles, the higher the energy, the lower stability of the system will result.

**Figure 3:**
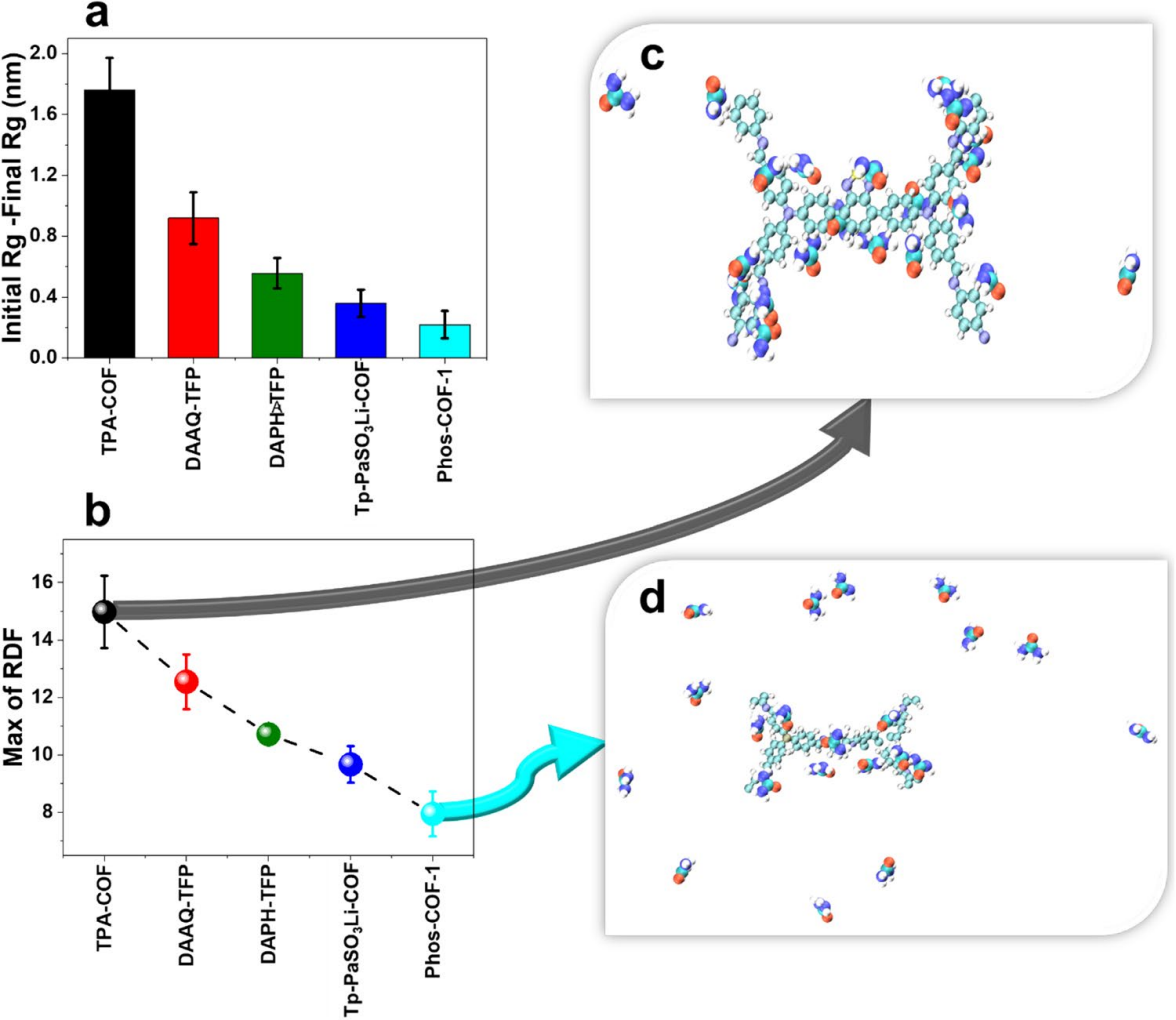
(**a**) The difference between initial and final gyration radius (**b**) Max RDF values for each system **(c**) TPA-COF nanoparticles with highest max of RDF (**d**) PhOS-COF-1 nanoparticles with lowest max of RDF.

In the current study, the difference between initial and final gyration radius is plotted in Fig. 3a. According to the diagrams, ${R}_{g}$ is decreased for all cases, which means that urea molecules aggregation occurred in the system. This change indicates that the urea molecules reach relatively stable circumstances, and the presence of intermolecular forces leads to forming a more stable and compact configuration. According to the results shown in Fig. 3a, the decrease of ${R}_{g}$ of urea molecules in the presence of the TPA-COF adsorbent was higher than other systems and it shows that TPA-COF was the more stable adsorbent for urea molecules. Furthermore, in other cases, urea molecules still had less tendency to disperse in the water as the molecules' aggregation has increased during the simulation. It should be mentioned that among all introduced adsorbents, no system was experienced a fluctuation in which the system loses its stability in comparison with its initial state, and this proves that all the adsorbents can be utilized in WAK devices. However, their adsorption quality determines the most efficient adsorbent.

#### Understanding the strength of interactions: radial distribution function (RDF)

The radial distribution function (RDF) or pair correlation function has been calculated to describe the strength of the interactions between the urea molecules and adsorbents through q the probability of finding urea molecules at a spherical shell of a certain thickness at a distance (r) from the surface of the nanoparticles^39^. In this analysis, the location of the first peak in the g(r) plot versus distance (r) signifies the intensity of urea molecules adsorption in which the higher maximum value represents the presence of more urea molecules around nanoparticles, indicating more significant adsorption.

Figure 3b exhibits the Max of RDF values of the urea molecules in the presence of various adsorbents. As can be observed, the highest RDF value is achieved when the TPA-COF has been used as an adsorbent. As previously mentioned, a significant accumulation of urea molecules is revealed by the maximum RDF value. Thus, the higher value of RDF for the TPA-COF can be ascribed to better adsorption. According to previous results indicating the stronger VdW interactions and the high amount of H-bonds in the system including the TPA-COF, occurring proper adsorption for this case is acceptable. Figure 3c,d illustrate the systems for the highest and lowest max of RDF values, respectively.

#### The capacity of sorbents: percentage of adsorption

To understand the adsorption rate of nanoparticles, we compared the total adsorbed urea molecules on the nanoparticle at the end of the simulation with the system's initial statues of present urea molecules. The results of this analysis showed that, all simulated nanoparticles have enough capability in urea molecules adsorption. However, the highest percentage of urea molecules adsorption was achieved with TPA-COF adsorbent (90%) followed by DAAQ-TFP (75%), DAPH-TFP (60%), Tp-PaSO_3_Li-COF (50%), and PhOS-COF-1 (40%). As was mentioned, 20 urea molecules were present in the simulation box. In the case of TPA-COF adsorbent, 18 urea molecules were adsorbed on the nanoparticle. This means that the TPA-COF has more suitable properties for utilization in WAK devices for removing urea molecules. It is worth noting that the decrease in percent adsorption of nanoparticle confirmed the results of the previous analysis.

### Coarse-grained and DFT simulations

In order to further elucidate the behavior of urea adsorption by the nanoparticles, coarse-grained simulations were performed. The length and time scale of MD simulations are their most significant barrier that limits MD application. The coarse-grained method can improve the simulation scale to mesoscale and catch the nanoscale behavior simultaneously by simplifying some unnecessary details of MD simulations^40^. We conducted a series of CG simulations with some of our previous analysis. Such theoretical information can help to better understand the capacity and mechanism of urea adsorption by these nanoparticles.

**Figure 4:**
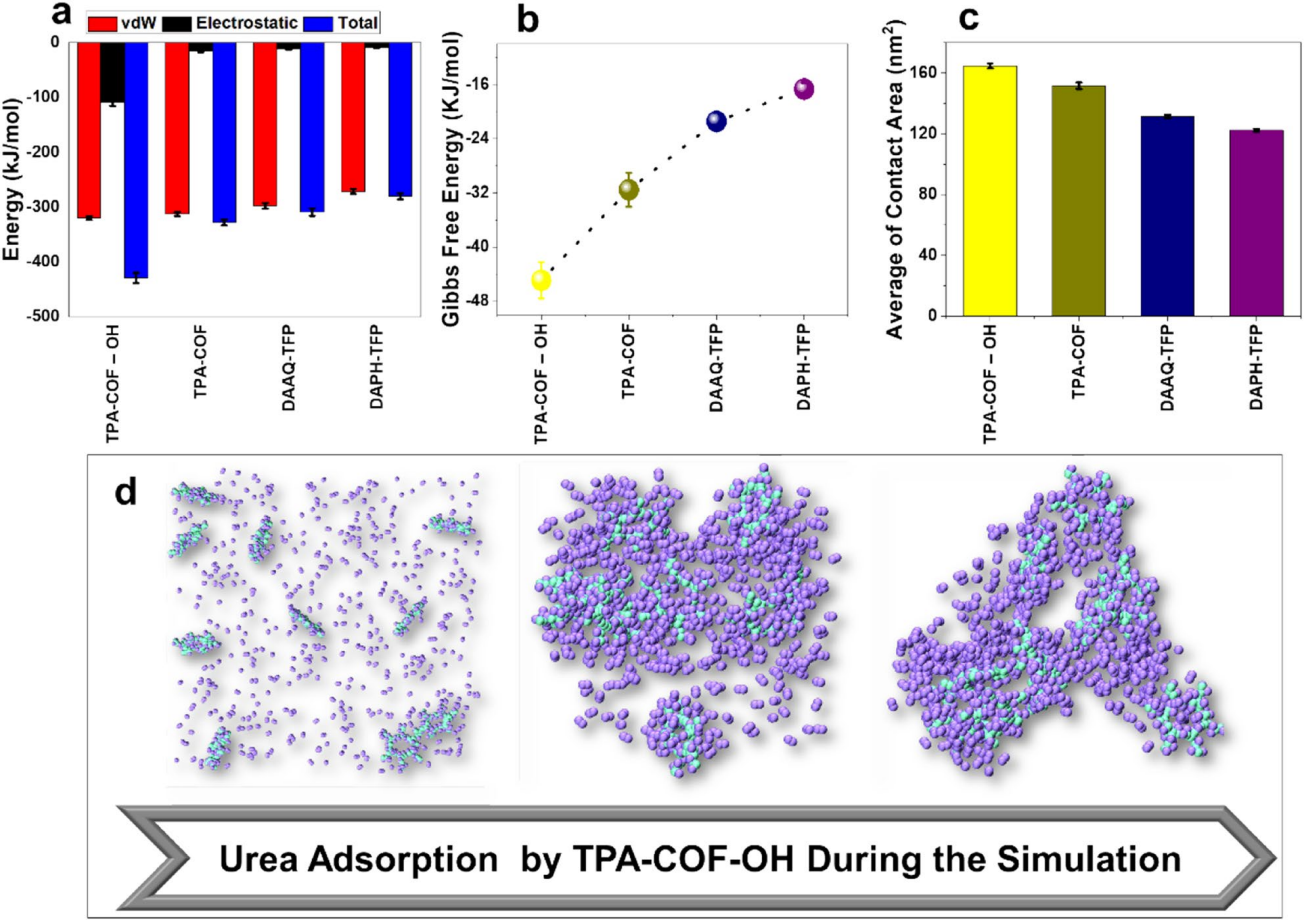
Coarse-grained simulation results. (**a**) Interaction energy (**b**) Gibbs free energy (**c**) Average of contact area (**d**) Process of urea adsorption by TPA-COF-OH during the simulation.

**Figure 5:**
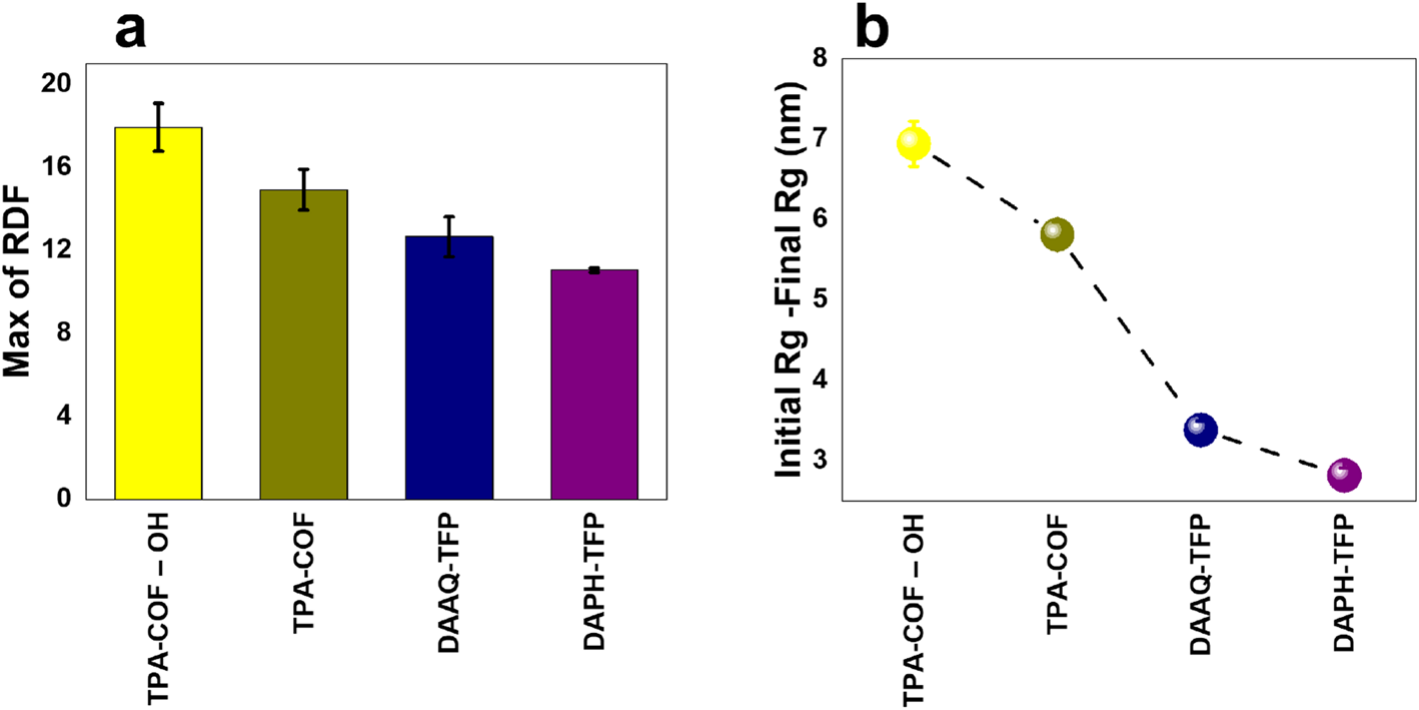
(**a**) The difference of initial and final gyration radius (**b**) The maximum of RDF values for each nanoparticle.

Moreover, we functionalized TPA-COF (best sorbent based on MD analysis) with –OH. Figures 4 and 5 show the CG results. In Fig. 4a, we can see that TPA-COF-OH has the highest total energy among all the nanoparticles. Although TPA-COF-OH and TPA-COF both have almost equal VdW energy, the difference in electrostatic energy makes the earlier nanoparticle a more suitable sorbent. Before modifying the surface of structure TPA-COF with hydroxyl functional group, its charge was zero. Therefore, interactions between this structure and urea molecules have been VdW interactions. But after functionalizing TPA-COF, the charge become negative. This has increased the electrostatic interactions between the TPA-COF-OH and the urea molecules. As a result, the electrostatic energy obtained from the adsorption of urea molecules on the TPA-COF-OH surface has increased significantly.

Figure 4b depicts the Gibbs free energy for different processes. Like the interaction energy analysis, these results, indicate that TPA-COF-OH has the more stable process of urea adsorption. For inspection of the possibility and condition of adsorption of urea molecules and each nanoparticle, the average contact area was analyzed (Fig. 4c). As can be seen, TPA-COF with –OH functional group has a higher average of contact area, and therefore, more suitable condition and the probability of urea adsorption. Figure 4d shows the process of urea adsorption by TPA-COF-OH nanoparticle during the simulation. The results of the two other analyses are shown in Fig. 5. In Fig. 5a, the difference of initial and final gyration radius for each nanoparticle is depicted. The TPA-COF-OH has the highest amount among all the nanoparticles. This implies that it has higher adsorption of urea molecules than other nanoparticles. Moreover, the maximum of RDF approves the previous results, and TPA-COF-OH shows a high capability of urea adsorption (Fig. 5b).

**Figure 6:**
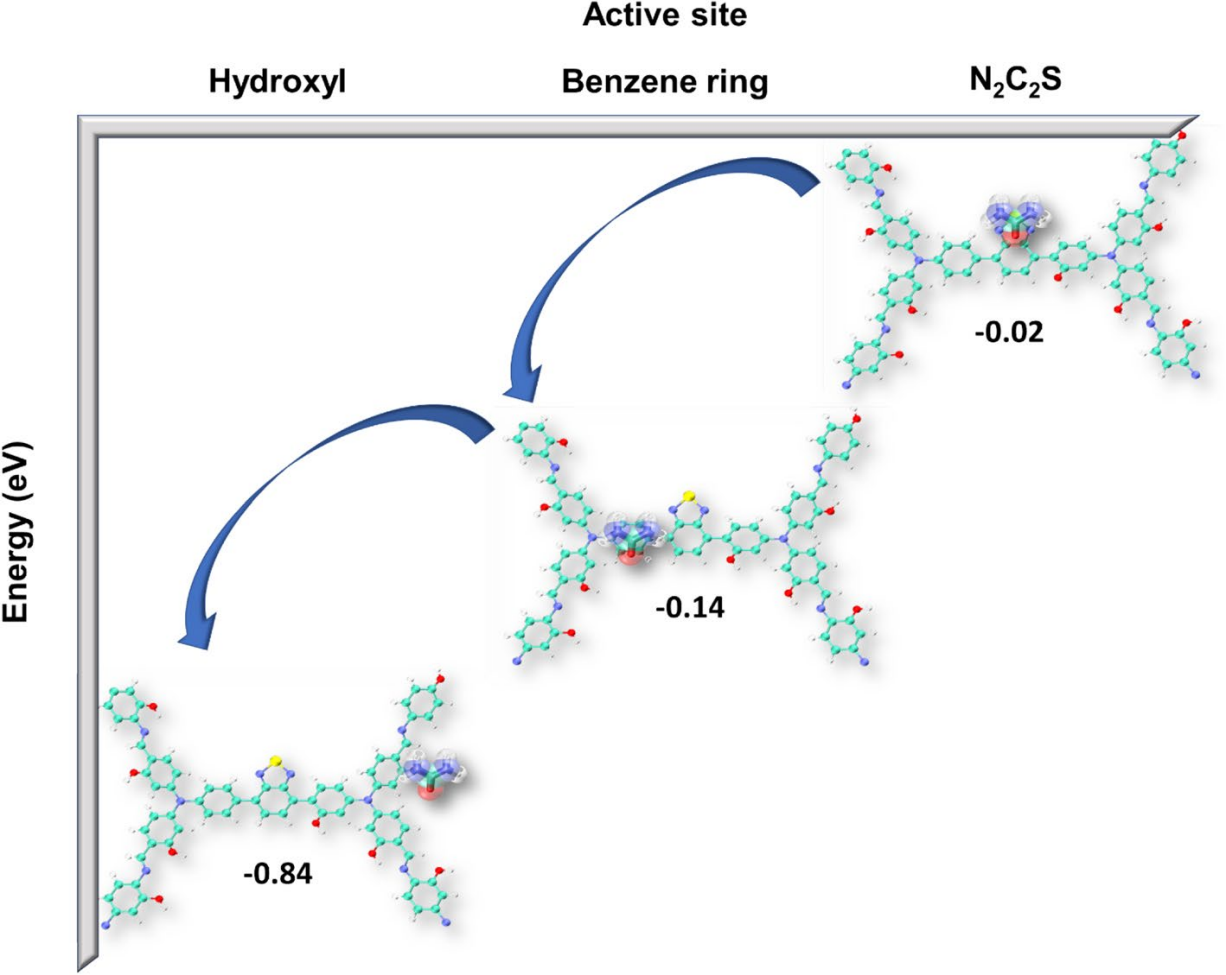
The adsorption energy of urea by TPA-COF-OH computed by DFT calculation.

All analyses follow the same result that TPA-COF-OH has the best characteristics to be an excellent sorbent for urea removal in the WAK devices. This can be due to interactions between the negative charge on the –OH functional group of TPA-COF-OH nanoparticle and the positive charge on the amine functional group of urea molecules. We used DFT calculation to compute the adsorption energy of urea by TPA-COF-OH. In this regard, the best site for urea adsorption can be obtained using DFT simulation. Figure 6 shows the interaction energy of the urea molecule and the different sites of the TPA-COF-OH. According to the obtained results, the interactions of urea and hydroxyl had the highest energy. After the hydroxyl site, the benzene ring site had the highest energy due to the interaction of urea and nanostructure. This indicates the tendency of urea adsorption by the hydroxyl and benzene ring sites. Also, high adsorption energy of this sites showed there was stability during the adsorption process.

### Investigation of urea adsorption in different pH

**Figure 7:**
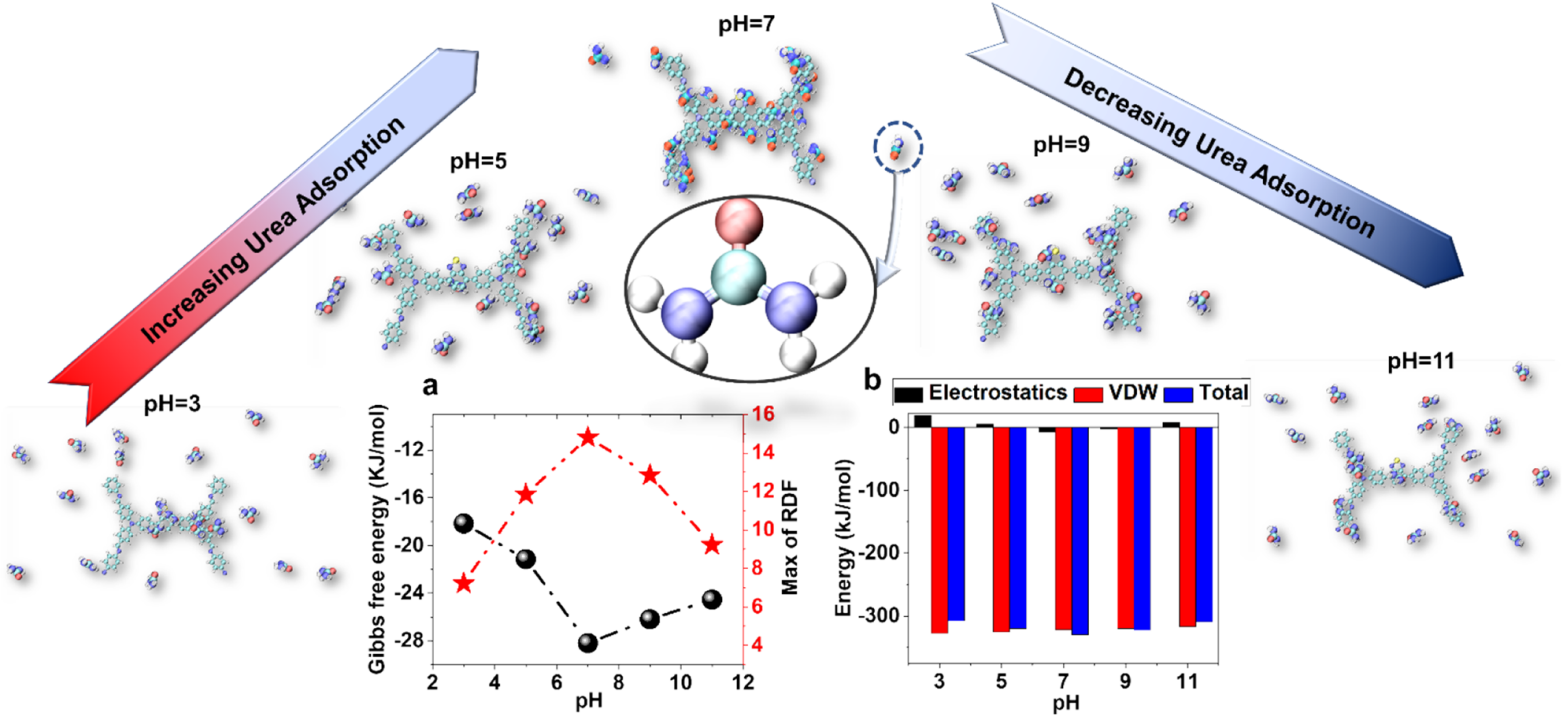
(**a**)The Gibbs free energy and maximum RDF of urea adsorption on the surface of TPA-COF in the different pH. (**b**)The adsorption energy of urea on the surface of TPA-COF in the different pH.

In this section, the intensity of urea adsorption in different pH is investigated. To do this, the best nanostructure is used to adsorb urea molecules. Figure 7b shows the amounts of energy from urea and TPA-COF interactions. Although, as in the previous results, the VDW interactions are dominant with the change of pH, the electrostatic interactions have increased. This is due to the increase in charge due to changes in the pH of the system. On the other hand, changing the pH has reduced the attraction between urea and TPA-COF. This is due to the increased repulsion force due to the increase of particles with a similar charge. Also, according to the results of the analysis, the electrostatic repulsion energy between urea and TPA-COF in acidic medium was more than in alkali medium.

In this regard, the results of RDF analysis are shown in Fig. 7a. Increased repulsion of particles with similar charges in alkali and acidic medium has reduced the density of urea molecules around TPA-COF. However, the adsorption of urea molecules in the acidic medium was less than the alkali medium, the urea adsorption in the alkali medium also lower than the adsorption of urea by TPA-COF in neutral medium.

In the following, the stability of urea adsorption in different pH is investigated. In this regard, Gibbs free energy due to urea adsorption on TPA-COF surface is shown in Fig. 7a. According to the obtained results, changing the pH of the system has reduced the amount of Gibbs free energy. In fact, changing the pH has made the urea adsorption on the TPA-COF structure unstable.

## Discussion

WAK devices with the urea adsorption process mechanism have been introduced as a promising method to overcome ESRD. However, the major challenge is the low efficiency of physical sorbents. In this study, the urea removal adsorption capacity of five types was investigated by applying molecular simulations tools. To this end, analyses such as Gibbs free energy, interaction energy, H-bonding, the radius of gyration (Rg), Solvent Accessible Surface Area (SASA), adsorption rate, radial distribution function (RDF), root mean square deviation (RMSD), and root-mean-square fluctuation (RMSF) have been carried out for AA simulations. The simulation results showed that although all of the COF NSs adsorbed urea molecules, the most suitable ones for the adsorption of urea molecules is TPA-COF. The results of Gibbs free energy, energy interaction, and the number of H-bonds illustrated the strongest attraction of urea molecules with TPA-COF, DAAQ-TFP, DAPH-TFP, Tp-PaSO_3_Li-COF, and PhOS-COF-1, respectively. The highest decrease in *Rg* was observed in TPA-COF, indicating more complex aggregation and compactness and adsorption process stability. Furthermore, the assessment of SASA analyses and adsorption rate of COF NSs confirmed the superior adsorption capacity of TPA-COF. Regarding RDF analysis, evaluation of the distribution of urea molecules around the NSs revealed that the closest accumulation of urea molecules was created around TPA-COF. Ultimately, to investigate the fluctuation of involved particles in the simulations, RMSD and RMSF analyses were conducted and the results showed that in the case of TPA-COF, the most stable urea molecules adsorption process occurred. In contrast, the worst stability was ascribed to PHOS-COF. Furthermore, a series of CG and DFT simulations were performed to capture the behavior of urea adsorption on a larger scale. In these simulations, one additional material was considered. The TPA-COF nanoparticles were modified with the –OH functional group, and the results showed that it has even better performance in urea adsorption than TPA-COF itself. To recapitulate, the AA analyses demonstrated that TPA-COF is the most effective NS to adsorb urea molecules, and CG analyses showed that TPA-COF-OH is even more useful. These two sorbents can positively improve the urea adsorption and make WAK devices feasible in confronting ESRD.

## Materials and methods

### System preparation

The molecular structures of TPA-COF, DAAQ-TPA, DAPH-TPA, Tp-PaSO_3_Li-COF, and PhOS-COF-1 were downloaded from the https://www.materialscloud.org/discover/curated-cofs#mcloudHeader. To compute charge distribution of atoms, the density functional theory (DFT) method was used and the B3LYP function has been applied in combination with the 6-31G + basis set. The number of atoms in COF NSs is indicated in Supplementary Table S1 and the initial structures of NSs are also illustrated in Supplementary Fig. S4. (Fig. 3 of the supplementary) Each MD simulation performed in a 6 nm length cubic box and a periodic boundary condition applied in all direction. It should be noted that in each simulation the box consists of one COF NS, 20 urea molecules, and 6500 water molecules.

### Computational details

All-atom (AA) molecular dynamics simulations were performed with the GROMACS simulation package^41^. The selected force field for AA in the simulation system is the OPLSS-AA force field, and the SPC/E model was used for water molecules as solvent^42^. The simulation initially started with energy minimization by the steepest descent method to remove any bad contacts between atoms for each run. The system was then allowed to equilibrate for 500 ps in canonical (NVT) ensemble followed by 500 ps in isothermal-isobaric (NPT) ensemble to achieve a stable system. The temperature and pressure were maintained constant at 300 K and 1 bar using V-rescaling and Parrinello–Rahman method, respectively^43–46^. After obtaining an equilibrium state, 30 ns MD simulation with one fs time step was performed. It should be noted that the Nose–hoover method was applied to keep the temperature constant in the production run^47,48^. Furthermore, all hydrogen bonds were constrained by the Linear Constraint Solver (LINCS) algorithm^49^. The long-range electrostatic interaction was calculated with the particle-mesh Ewald summation method^50^. The cut-off distances of 1.4 nm were taken for both Van der Waals interactions and Coulombic interactions.

For coarse-grained (CG) simulation, it has been done with GROMACS 2020 software in coarse-grained mode and with Martini force field^51,52^. The structures of COFs were extracted from the Material cloud site. Then the esp charge and their topology are obtained through DFT calculations and the PolyParGen online server. The structural files were then converted to Martini force field format using Avogadro software. The Martini force field parameters were also calculated through the Martini site, and reference article, and the topology file was modified using the obtained parameters. Coarse-grained simulations were optimized with an energy tolerance of 100 kJ/mol. Then the simulation box reached temperature and pressure equilibrium during 3 stages of equilibrium with different time steps of 0.01 fs, one fs, and 20 fs and a time of 3 ns. The algorithms used in these steps were Nose Hoover and Parrinello-Rahman. Coulomb energy calculation algorithm was also PME (Particle Mesh Ewald). The cut-off radius for Van der Waals and electrostatic interactions was 3 nm, and the simulation was performed at 1000 ns with time steps of 20 fs.

DFT simulation was performed using Gaussian software in which urea molecules were placed in two separate simulations at a distance of 30 angstroms from the TPA-COF-OH surface. Then, the simulation with the basis set 6-31++G and B3LYP method was performed and the energy between the TPA-COF-OH and urea was calculated.

### Umbrella sampling method

The urea adsorption simulation was performed in a 6 × 6 × 12 nm box for 30 ns at 1 fs time steps. Then, by using the Umbrella sampling, we calculated the Gibbs free energy. So, after setting the isothermal–isobaric (NPT) ensemble at 1000 ps, using the pull code method, the urea molecule was isolated from the COFs. Regarding this, the urea molecule was stretched along the z-axis by 6 nm. Then, at 0.06 nm intervals, 100 configurations were extracted. Finally, using Wham analysis on the configurations results, the Gibbs free energy of urea adsorption was calculated.

## Supplementary Information

Supplementary Information.
