# Supplementary material for: Molecular insight into COF monolayers for urea sorption in artificial kidneys
Source: Sci Rep. 2021 Jun 8;11:12085. doi: 10.1038/s41598-021-91617-1 (PMC8187635; doi:10.1038/s41598-021-91617-1)
Supplement: Supplementary file 1 — Supplementary Information. [file 41598_2021_91617_MOESM1_ESM.docx]

**Molecular Insight into COF Monolayers for Urea Sorption in Artificial Kidneys**

Ahmad Miri Jahromi^a^, Mohammad Khedri^a^, Mehdi Ghasemi^a,b^ ,Sina Omrani^c^, , Reza Maleki^a,*^, Nima Rezaei^d,e,f*^

*^a^ Computational Biology And Chemistry Group (CBCG), Universal Scientific Education and Research Network (USERN),Tehran, Iran*

*^b^ Department of Petroleum Engineering, Amirkabir University of Technology, Tehran, Iran*

*^c^ Department of Petroleum Engineering, University of Tehran, Iran*

*^d^ Research Center for Immunodeficiencies, Pediatrics Center of Excellence, Children’s Medical Center, Tehran University of Medical Sciences, Tehran, Iran*

*^e^ Network of Immunity in Infection, Malignancy and Autoimmunity (NIIMA), Universal Scientific Education and Research Network (USERN), Tehran, Iran*

*^f^ Department of Immunology, School of Medicine, Tehran University of Medical Sciences, Tehran, Iran*

^*^ Corresponding Authors: Prof. Nima Rezaei *rezaei_nima@tums.ac.ir* and Dr. Reza Maleki


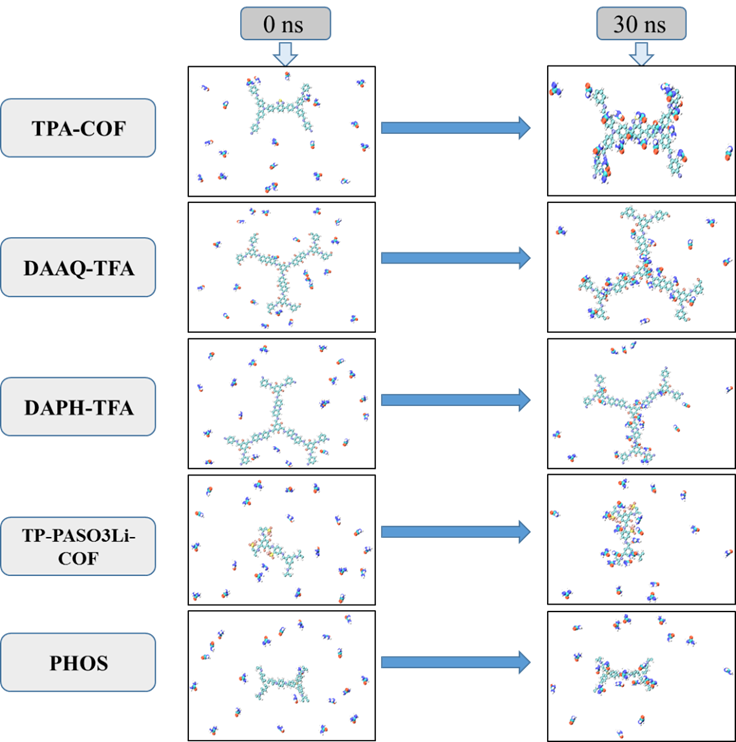


Figure. S1. Spatial position of the urea molecules at the beginning and end of the simulation in presence of different sorbents

**Studying the stability of system: The Root-Mean-Square Deviation (RMSD)**

This analysis provides insight into the deviation of the position of particles relative to their reference position at each time .RMSD is considered an analysis to assess a system's stability, while MD simulation in which the narrower RMSD range represents the more stability of a system ^5,6^.

Figure. 5a illustrates the average of RMSD values of urea molecules in the presence of various adsorbents. The high fluctuation of a particle in the optimization phase of simulation created a sudden surge in RMSD. As simulation proceeds, different involved particles oscillate to achieve their most stable condition during the simulation. That is why particle fluctuation during the simulation is considered a perfect indicator to assess the simulation system's stability. To reach relative stability, particle fluctuation should be decreased, and the slope of the RMSD diagram is reduced to zero until the end of the simulation process. This can be seen in Figure S2.a. However, for other simulated systems the particle fluctuations exist which indicates the relative instability of simulation systems.

**Studying the stability of system: The root-mean-square fluctuation (RMSF)**

This analysis measures the average deviation of a particle over simulation time from a reference position which is considered the particle's time-average position.

Unlike the RMSD analysis that indicates the positional difference between an entire structure over time, RMSF analyzes a particular atom's fluctuation during the simulation. In this analysis, the higher RMSF values specify more instability of the system ^7-9^. The average RMSF values for all atoms are shown in Figure. S4.b As similar to the previous analysis, RMSD, the minimum fluctuation occurred in the TPA-COF and DAAQ-TFP. Systems that include the mentioned adsorbents experienced fewer oscillations. Thus, stable conditions are present during the adsorption process of urea molecules on the adsorbents.


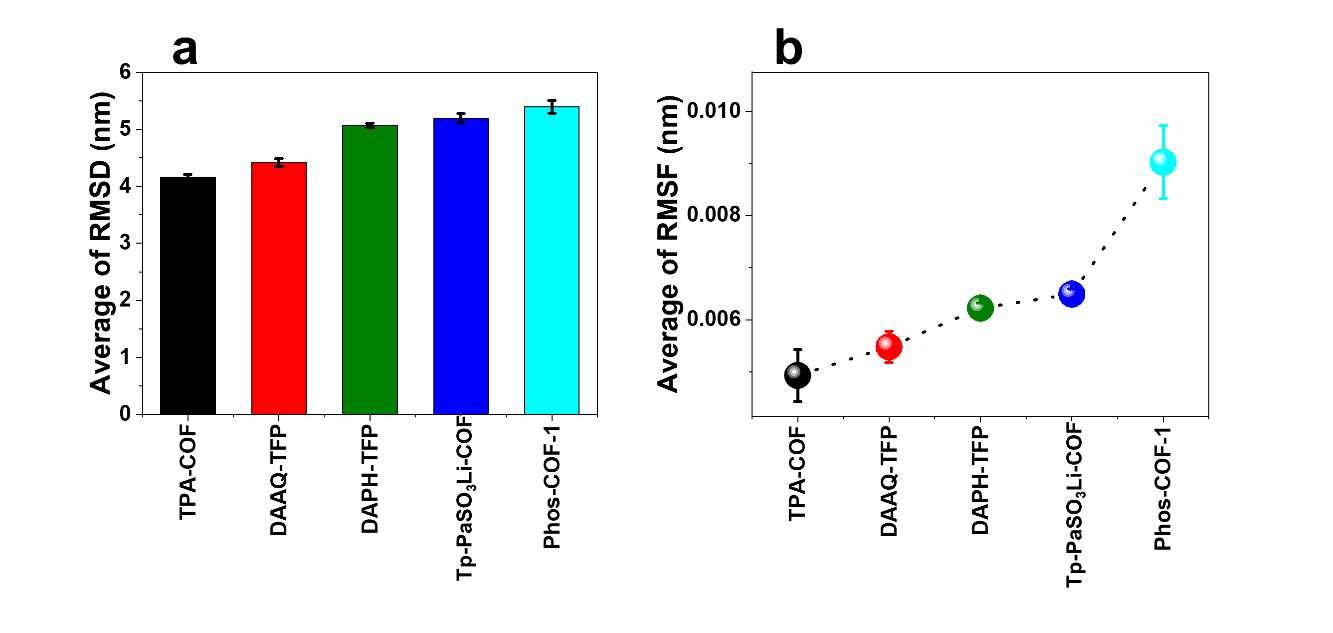


Figure. S2. (**a**) Average of RMSD values (**b**) Average of RMSF values


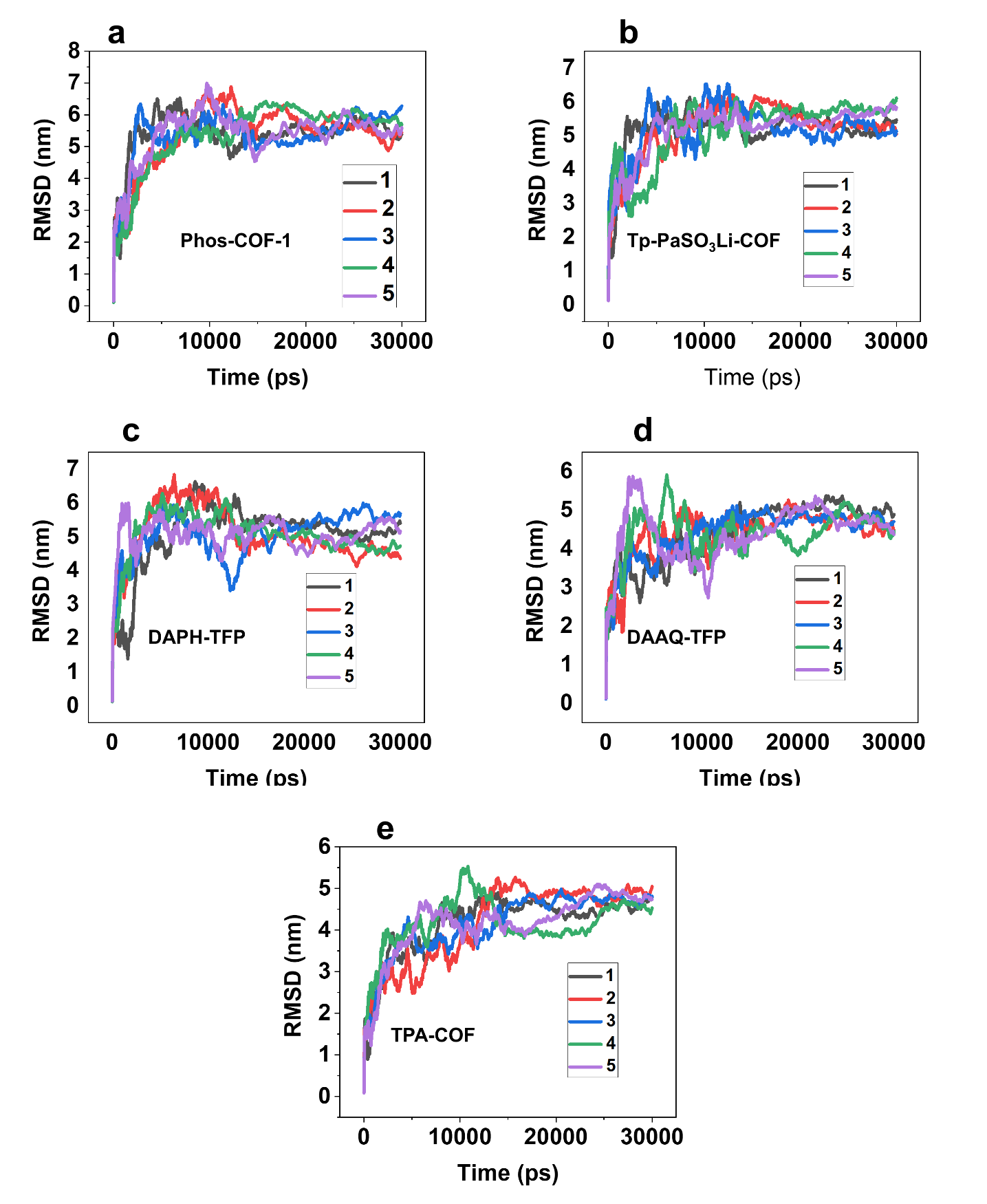


Figure. S3. (**a-e)** RMSD value of urea in the presence of the nanostructures for every five repetitions.


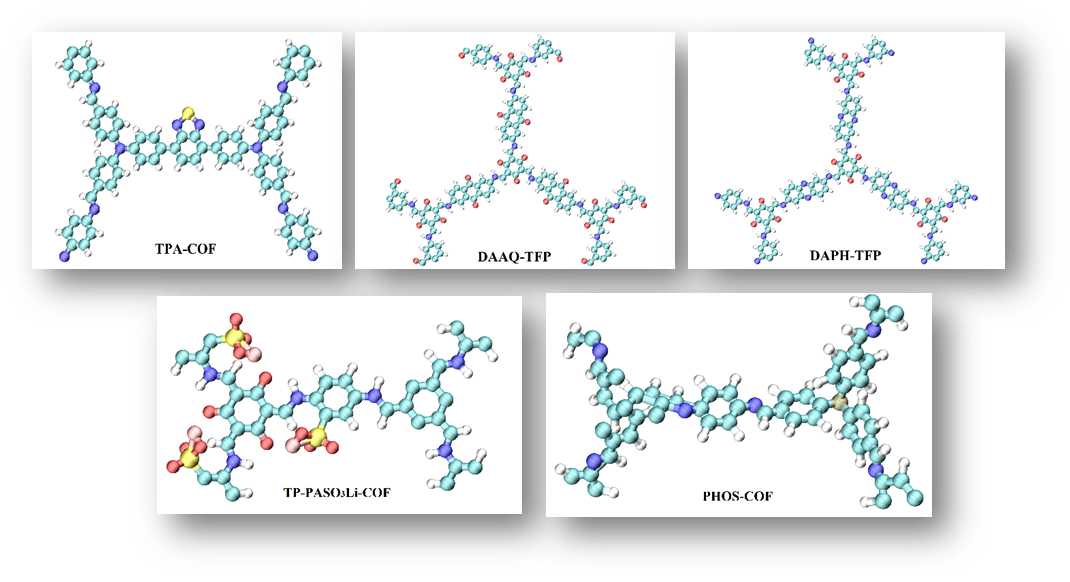


Figure. S4. The snapshots of COF NSs.

Table S1.Number of atoms in each invidiual COF NSs.

| Molecule Name | Chemical Formula |
| --- | --- |
| TPA-COF | C_70_H_52_N_10_S |
| DAAQ-TPA | C_120_H_84_N_12_O_24_ |
| DAPH-TPA | C_108_H_84_N_24_O_12_ |
| TP-PASO3Li-COF | C_36_H_52_N_6_S_3_ |
| PHOS-COF | C_57_H_79_N_6_P |

**Reference**

1 Ungerer, P., Nieto-Draghi, C., Rousseau, B., Ahunbay, G. & Lachet, V. Molecular simulation of the thermophysical properties of fluids: From understanding toward quantitative predictions. Journal of Molecular Liquids 134, 71-89 (2007).

2 Verlet, L. Computer" experiments" on classical fluids. I. Thermodynamical properties of Lennard-Jones molecules. Physical review 159, 98 (1967).

3 Gear, C. W. Numerical initial value problems in ordinary differential equations. Prentice-Hall series in automatic computation (1971).

4 Swope, W. C., Andersen, H. C., Berens, P. H. & Wilson, K. R. A computer simulation method for the calculation of equilibrium constants for the formation of physical clusters of molecules: Application to small water clusters. The Journal of chemical physics 76, 637-649 (1982).

5 Saini, R. K., Thakur, H. & Goyal, B. Effect of Piedmont mutation (L34V) on the structure, dynamics, and aggregation of Alzheimer’s Aβ40 peptide. Journal of Molecular Graphics and Modelling 97, 107571 (2020).

6 Rahmatabadi, S. S., Sadeghian, I., Ghasemi, Y., Sakhteman, A. & Hemmati, S. Identification and characterization of a sterically robust phenylalanine ammonia-lyase among 481 natural isoforms through association of in silico and in vitro studies. Enzyme and microbial technology 122, 36-54 (2019).

7 Chairatana, P., Niramitranon, J. & Pongprayoon, P. Dynamics of human defensin 5 (HD5) self-assembly in solution: Molecular simulations/insights. Computational biology and chemistry 83, 107091 (2019).

8 Rezapour, N. et al. Molecular dynamics studies of polysaccharide carrier based on starch in dental cavities. International journal of biological macromolecules 121, 616-624 (2019).

9 Liu, J. et al. Enhanced stability of manganese superoxide dismutase by amino acid replacement designed via molecular dynamics simulation. International journal of biological macromolecules 128, 297-303 (2019).
